# Molecular Research and Treatment of Urologic Cancer

**DOI:** 10.3390/ijms252413666

**Published:** 2024-12-20

**Authors:** Hironobu Yamashita

**Affiliations:** Department of Pathology and Laboratory Medicine, Pennsylvania State University College of Medicine, Hershey, PA 17033, USA; hyamashita@pennstatehealth.psu.edu

Urologic cancers exhibit tumor heterogeneity in their morphology, gene expression, and phenotypic features, and have different responses to drug treatment [1]. Thus, it is significant to elucidate the molecular mechanisms of heterogeneity to overcome this therapeutic problem for cancer patients.

Therefore, this Special Issue focuses on urologic cancers. Five research articles and one review article were collected in the Special Issue “Molecular Research and Treatment of Urologic Cancers” to understand the molecular mechanisms of prostate, renal (kidney), and bladder cancer.

(1) Prostate Cancer Research

Filippi et al. published a research article titled “Analysis of the gene networks and pathways correlated with tissue differentiation in prostate cancer”, which tried to correlate gene expression with the Gleason score of prostate cancer. Using The Cancer Genome Atlas (TCGA), a publicly available database of 497 prostate cancer specimens, the authors reported that high Gleason scores were linked to sensitivity to DNA damage, cell division, and telomere elongation. Their findings contribute to a deeper understanding of how gene expression relates to the progression and aggressiveness of PCa [2].

Paulus et al. published a research article titled “The effect of photosensitizer metalation incorporated into arene–ruthenium assemblies on prostate cancer”. The various treatments for prostate cancer have several side effects. However, photodynamic therapy (PDT) has almost no adverse effects. This method uses a combination of photosensitizers and light to kill cells by inducing the generation of reactive oxygen species inside cells. This study investigated the role of metals, including magnesium, cobalt, and zinc, in photosensitizers used in this therapeutic method. The authors demonstrated that these metals can induce destructive effects in prostate cancer cell lines. However, the addition of metals to photosensitizers reduces the efficacy of PDT [3]. 

(2) Renal Cancer Research

Two research articles on renal cancer have been published. 

Jedrzejewska et al. published a research article titled “Elevated plasma concentration of 4-pyridone-3-carboxamide-1-β-D-ribonucleoside (4PYR) highlights the malignancy of renal cell carcinoma”.

Nicotinamide (NA) is a precursor compound of 4PYR that plays a significant role in multiple biological functions encompassing DNA repair mechanisms, cell cycle progression, and inflammatory processes. 4PYR was previously reported by these authors to be an oncometabolite in breast, lung, and bladder cancer. The authors reported that 4PYR could be an oncometabolite in the clear cell renal cell carcinoma (ccRCC) subtype and that elevated levels of 4PYR are highly correlated with tumor aggressiveness, mortality, recurrence, and metastasis in ccRCC patients. Their results showed 4PYR’s potential as an oncometabolite in the ccRCC subtype [4].

Larrinaga et al. published a research article titled “The Expression of Alamandine Receptor MrgD in Clear Cell Renal Cell Carcinoma Is Associated with Worse Prognosis and Unfavorable Response to Antiangiogenic Therapy”.

The variability in renin–angiotensin system elements within the kidney impacts patient prognosis and therapeutic responsiveness. Alamadine (ALA) represents a newly discovered component of the renin–angiotensin system inside the kidney. In this study, we investigated the role of MrgD in ccRCC. This study revealed that high levels of MrgD expression were highly related to poor prognosis among ccRCC patients and decreased responsiveness to antiangiogenic therapy using tyrosine kinase inhibitors. This suggests that MrgD could be a potential biomarker for prognosis and for the assessment of response to treatment in patients with ccRCC [5]. 

(3) Bladder Cancer Research

Kishi et al. published a research article titled “ERVK13-1/miR-873-5p/GNMT Axis Promotes Metastatic Potential in Human Bladder Cancer through Sarcosine Production”. Sarcosine (N-dimethylglycine) is an oncometabolite that facilitates the metastatic ability of cancers. Sarcosine is generated from glycine via the Glycine-N-methyltransferase (GNMT) enzyme. The authors elucidated the molecular mechanisms of GNMT regulation via microRNA and lncRNA in sarcosine production, which is related to the aggressiveness of bladder cancer. Furthermore, the authors concluded that sarcosine concentration in urine may be used as a biomarker in patients with bladder cancer [6]. 

Gill et al. published a mini-review article titled “Current Bladder Cancer Treatment-The Need for Improvement”. Bladder cancer lacks treatment options, whereas advancements have been observed in other cancers. These limited treatment options lead to high costs and a poor quality of life. In this study, the authors described some promising approaches, including urinary biomarkers, targeted therapies such as the PI3K-Akt-mTOR pathway, immune checkpoint inhibitors (PD-1 and CDLA-4 antibodies), and drug repositioning, which could overcome these barriers [7]. 
